# *CHEK2* variants associate with hereditary prostate cancer

**DOI:** 10.1038/sj.bjc.6601425

**Published:** 2003-11-11

**Authors:** E H Seppälä, T Ikonen, N Mononen, V Autio, A Rökman, M P Matikainen, T L J Tammela, J Schleutker

**Affiliations:** 1 Laboratory of Cancer Genetics, Institute of Medical Technology, Lenkkeilijänkatu 8,University of Tampere and Tampere University Hospital, FIN-33014 University of Tampere, Finland; 2 Research Unit, Tampere University Hospital, FIN-33521 Tampere, Finland; 3 Department of Urology, Tampere University Hospital and Medical School, University of Tampere, FIN-33521 Tampere, Finland

**Keywords:** prostate cancer, checkpoint, CHEK2, 1100delC, I157T

## Abstract

Recently, variants in *CHEK2* gene were shown to associate with sporadic prostate cancer in the USA. In the present study from Finland, we found that the frequency of 1100delC, a truncating variant that abrogates the kinase activity, was significantly elevated among 120 patients with hereditary prostate cancer (HPC) (four out of 120 (3.3%); odds ratio 8.24; 95% confidence interval 1.49–45.54; *P*=0.02) compared to 480 population controls. Suggestive evidence of segregation between the 1100delC mutation and prostate cancer was seen in all positive families. In addition, I157T variant had significantly higher frequency among HPC patients (13 out of 120 (10.8%); odds ratio 2.12; 95% confidence interval 1.06–4.27; *P*=0.04) than the frequency 5.4% seen in the population controls. The results suggest that *CHEK2* variants are low-penetrance prostate cancer predisposition alleles that contribute significantly to familial clustering of prostate cancer at the population level.

Analyses using families with hereditary prostate cancer (HPC) have suggested that multiple genetic loci may harbour prostate cancer susceptibility genes, including HPC1 (MIM 601518) at 1q24–q25, HPC2 (MIM 605367) at 17p11, PCAP (MIM 602759) at 1q42–q43, HPCX (MIM 300147) at Xq27–q28, CAPB (MIM 603688) at 1p36, and HPC20 (MIM 176807) at 20q13 (Nwosu *et al*, 2001). So far, only two genes have been identified from these chromosomal regions: *ELAC2* from HPC2 –locus (Tavtigian *et al*, 2001) and *RNASEL* (MIM 180435) from HPC1 –locus (Carpten *et al*, 2002). In Finland neither *ELAC2* nor *RNASEL* did explain the disease segregation in HPC families, but seemed to have some kind of modifying role in prostate carcinogenesis (Rokman *et al*, 2001; Rokman *et al*, 2002). Xu *et al* (2001) identified a new locus at 8p22–23, and mutations in *MSR1* gene (MIM 153622) were reported to associate with prostate cancer (Xu *et al*, 2002). However, recent results do not support a major role for the *MSR1* gene in the causation of prostate cancer (Seppala *et al*, in press). Definitive confirmations of the role of *ELAC2*, *RNASEL*, or *MSR1* in prostate cancer predisposition are still warranted.

Recently, mutations in *CHEK2* (MIM 604373) were identified in patients with prostate cancer (Dong *et al*, 2003). The *CHEK2* gene localises to chromosome 22q12.1 and contains 14 exons. Originally, germline mutations in *CHEK2* gene were reported in Li–Fraumeni syndrome and breast cancer (Bell *et al*, 1999; Allinen *et al*, 2001). Rare somatic mutations in *CHEK2* have also been identified in a number of cancer types, including lung and ovarian cancers and osteosarcomas (Miller *et al*, 2002). These results together with the normal function of *CHEK2* in DNA damage checkpoints are consistent with the idea that *CHEK2* might act as a tumour suppressor gene. Here, we explored the significance of *CHEK2* gene in prostate cancer causation in Finland. The Finnish population is known to be historically isolated and genetically homogeneous (Peltonen *et al*, 2000). Therefore, there may be a limited number of prostate cancer causing mutations, and the effect of individual risk genes could be identified more readily than in more heterogeneous populations.

## MATERIALS AND METHODS

### Families with HPC

Collection of Finnish families with HPC has been described elsewhere (Schleutker *et al*, 2000). For single-strand conformation polymorphism (SSCP) analysis, youngest affected patient from each of the 120 HPC families was initially used for the screening of *CHEK2* mutations. The HPC families consisted of two cohorts. The families in the first cohort (*n*=68) had either three or more first- or second-degree- affected members or two affected members with at least the index patient diagnosed with prostate cancer ⩽60 years of age. The mean age at diagnosis for the index patients was 62.2 years (range 44–81 years), and the mean number of affected family members was 3.2 (range 2–6). The second cohort of families (*n*=52) had only two first- or second-degree affected members with ages at diagnosis >60 years. The mean age at diagnosis for the index patients was 69.0 years (range 61–86).

### Patients with prostate cancer and controls

The 1100delC and I157T variants were analysed in 537 patients with unselected prostate cancer, and in 480 healthy male blood donors. There were altogether 634 consecutive patients diagnosed with prostate cancer in the Pirkanmaa Hospital District with a population of around 450 000 during 1999–2000. We had samples from 85% of these patients, which results in an unselected, population-based collection of patients. The mean age at diagnosis for the patients with unselected prostate cancer was 68.6 years (range 47–90). Information was available on the tumour WHO-grade in 96%, on Gleason score in 88%, on T-stage in 100%, and on N-stage in 30% of the patients. M-stage was ascertained by bone scan in 73% of the patients with PSA⩾10 *μ*g l^−1^ and in 26% of the patients with PSA<10 *μ*g l^−1^. In all, 12% (66 out of 537) of the patients reported a positive family history of prostate cancer. The controls consisted of DNA samples from anonymous male blood donors obtained from the Blood Center of the Finnish Red Cross in Tampere.

Written informed consent was obtained from all living patients and also, for families with HPC, from the unaffected members. The research protocols were approved by the Ethical Committee of the Tampere University Hospital (93175, 95062, and 99228), and the National Human Genome Research Institute (HG-0158). Permission for collection of families, in the entirety of Finland, was granted by the Ministry of Health and Social Affairs (59/08/95).

### Mutation screening with SSCP analysis

Single-strand conformation polymorphism analysis of the entire coding sequence of the *CHEK2* gene was designed to include all intron–exon boundaries (GenBank accession number AF086904). Primers used for amplification of exons 10–14, which are known to be repeated on several other chromosomes (Sodha *et al*, 2000), were designed so that both primers for each primer pair had a base mismatch in the most 3′ nucleotide, compared with sequences from nonfunctional copies of *CHEK2*. Genomic DNA was used at 25 ng per 15 *μ*l reaction mixture containing 1.5 mM MgCl_2_; 20 *μ*M each of dNTP; 0.5 *μ*Ci of *α*(^33^P)-dCTP (Amersham Pharmacia, Uppsala, Sweden); 0.6 *μ*M of each primer; 1.0 U Ampli*Taq*Gold; and the reaction buffer provided by the supplier (PE Biosystems, Foster City, CA, USA). Annealing temperature of 50°C (for exons 10 and 12) or 55°C (for all other exons) was used. After denaturation, the (^33^P)-labeled PCR products were electrophoresed at 800 V for 12 h at room temperature, in 0.5 × mutation-detection-enhancement gel (FMC BioProducts, Rockland, ME, USA) with 1% glycerol in 0.5 × Tris-borate EDTA. For exon 11, the electrophoresis was also performed without glycerol in the gel. After electrophoresis, gels were dried and exposed to Kodak BioMax maximum-resolution films for 6 h. All samples, in which variant bands were detected, as well as two normal bands per exon, were sequenced using the same PCR primers and ABI Prism 310 Genetic Analyzer (PE Biosystems, Foster City, CA, USA). Also, the genotypes of the available family members of the mutation carriers were determined by sequencing.

### Minisequencing and SSCP for large-scale population screening of identified variants

The frequencies of the two *CHEK2* variants were determined in the entire sample of patients described above. 1100delC variant was screened by minisequencing (Syvanen, 1998). PCR was performed with 100 ng of DNA, 0.2 *μ*M each primer, 0.2 mM each dNTP, 1.5 mM MgCl_2_, and 1.0 U of Ampli*Taq*Gold (PE Biosystems, Foster City, CA, USA), in a final volume of 50 *μ*l. I157T variant was screened by SSCP analysis as described above. Positive results from both mutation analyses were confirmed by sequencing.

### Statistical analyses

Association of the CHEK2 genotypes with HPC and unselected prostate cancer was tested by logistic-regression analysis, by use of the SPSS statistical software package (SPSS 11.0). Association with demographic, clinical, and pathological features of the disease was tested by the Mann–Whitney test, Pearson *χ*^2^ test, and Fisher's exact test by use of the SPSS statistical software package (SPSS 11.0).

## RESULTS

Five sequence variants were identified in the SSCP analysis of the *CHEK2* gene in 120 index patients from Finnish families with HPC (Table 1

**Table 1:** Summary of *CHEK2* germline variants found in 120 patients with HPC in the SSCP analysis

**Mutation**	**Amino-acid change**	**Exon/intron**	**Domain**
252A>G	Silent (E84)	Exon 1	Unknown
319+(43−44)insA	—	Intron1	—
470T>C	I157T	Exon 3	FHA^a^
1100delC	Frameshift	Exon 10	Kinase
1312G>T	D438Y	Exon 11	Kinase

a FHA=forkhead-associated domain.

). Two of the variants, a missense variant 470T>C (I157T) in exon 3 and a frameshift mutation 1100delC in exon 10, were the same as previously reported in patients with Li–Fraumeni syndrome (Bell *et al*, 1999), breast (Allinen *et al*, 2001; Vahteristo *et al*, 2002), and prostate cancer (Dong *et al*, 2003). The frameshift 1100delC mutation has been proven to result in the loss of kinase activity (Wu *et al*, 2001), and I157T variant has been shown to be defective in its ability to bind and phosphorylate Cdc25A, one of its normal substrates (Falck *et al*, 2001). These variants were further studied in a set of 1137 samples. In addition, a silent exonic change also reported by Bell *et al* (1999), an intronic change (not affecting splice site), and a novel missense mutation 1312G>T (D438Y) were observed. D438Y mutation was found only in one proband. In this family there were two prostate cancer patients. Unfortunately, we did not have a sample from the second affected person (deceased).

The 120 patients with HPC included 3.3% (four out of 120) patients who carried the 1100delC mutation (Table 2

**Table 2:** Association of the 1100delC and I157T variants of the *CHEK2* gene with patients with unselected prostate cancer or HPC

**Mutation and sample**	**No. of carriers/total (frequency)**	**OR**	**95% CI**	***P***
*1100delC*				
Controls	2/480 (0.4%)	1.00		
Patients with unselected Prostate cancer	7/537 (1.3%)	3.14	0.65−15.16	0.15
Patients with HPC	4/120 (3.3%)	8.24	1.49−45.54	0.02^a^
				
*I157T*				
Controls	26/480 (5.4%)	1.00		
Patients with unselected Prostate cancer	42/537 (7.8%)	1.48	0.89−2.46	0.13
Patients with HPC	13/120 (10.8%)	2.12	1.06−4.27	0.04^a^

a Statistically significant.

). This was significantly higher (odds ratio (OR)=8.24; 95% confidence interval (CI) 1.49−45.54; *P*=0.02) than the frequency 0.4% seen in population sample of 480 blood donors. Among the unselected patients with prostate cancer, the frequency of 1100delC variant was 1.3% (seven out of 537). All 1100delC carriers were heterozygous. All other affected and unaffected male relatives were also genotyped from 1100delC-positive families. Suggestive evidence of segregation between the 1100delC mutation and prostate cancer was seen in all four families (Figure 1

**Figure 1:**
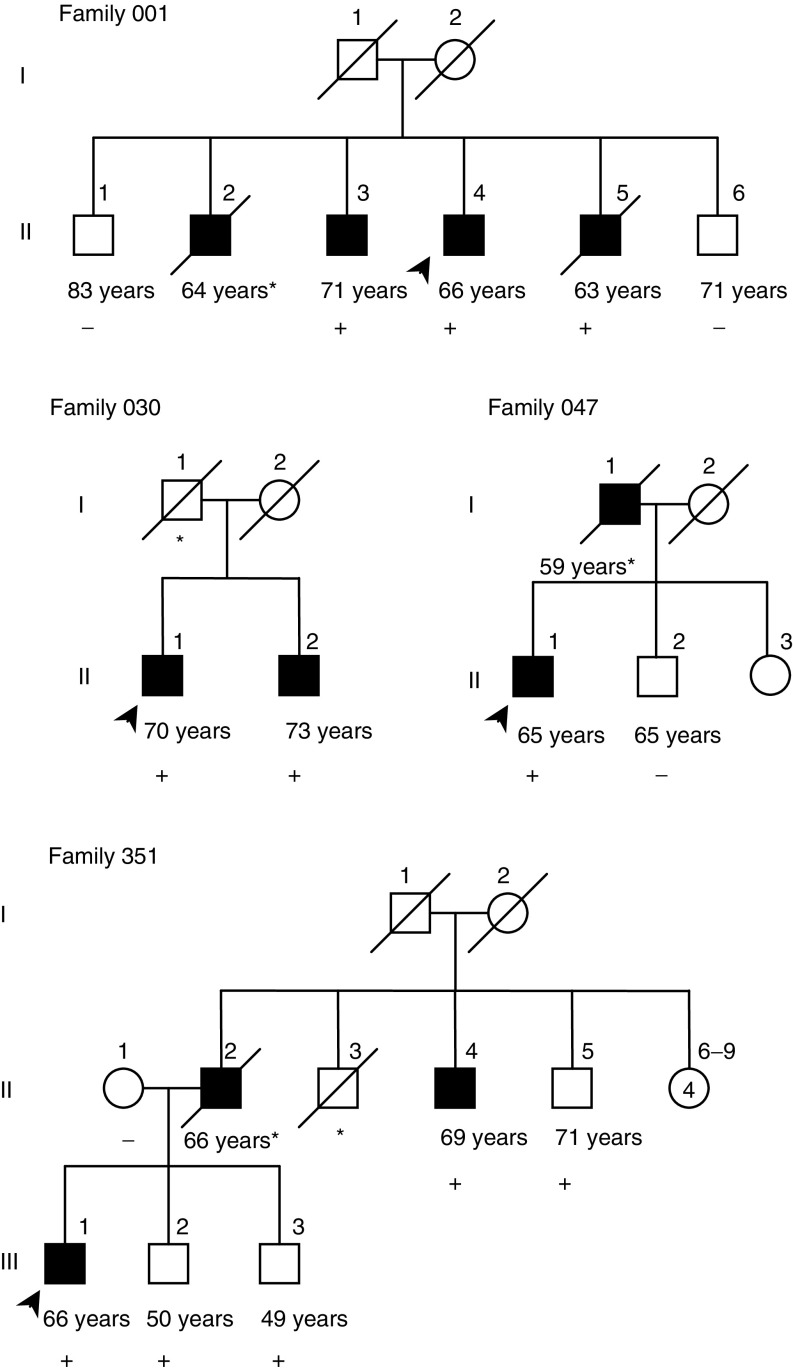
Segregation of *CHEK2* 1100delC mutation in four families with HPC. 1100delC variant carriers are denoted by a plus sign (+), and noncarriers by a minus sign (−). An asterisk (^*^)) denotes the persons with no sample available. No sample was available from affected father (II-2) in family 351, but because the mother (II-1) did not carry the mutation, the father is a likely 1100delC mutation carrier. Current age of the unaffected members or age at diagnosis for prostate cancer patients (in years) is indicated below the symbol for each family member. In each family, the index patient is marked with an arrow. Squares denote male subjects, and circles denote female subjects; black symbols denote patients with prostate cancer.

). Since 1100delC mutation has been reported in Li–Fraumeni syndrome and was previously called a mutation hot spot (*CHEK2* [MIM 604373]), we looked other cancers in these four 1100delC-positive families. In family 001, there was one second-degree relative diagnosed with lung cancer, but no other cancers were found among first- or second-degree relatives.

The I157T variant was seen in 10.8% (13 out of 120) of patients with HPC (Table 2). This was also significantly higher (OR=2.12; 95% CI 1.06−4.27; *P*=0.04) than the frequency of 5.4% seen in the population controls. Nine of the I157T-positive families had only two affecteds, three families had three affecteds, and one family had four affecteds. Segregation of the variant with the disease was incomplete, in that both unaffected mutation carriers and mutation-negative patients with prostate cancer were observed. In addition, the I157T variant was found in 7.8% (42 out of 537) of unselected prostate cancer patients. One of these carriers was homozygous; all other I157T variant carriers were heterozygous. The homozygous carrier did not have family history of cancer and there was nothing unusual in his phenotype. None of the patients or controls carried both 1100delC and I157T variant.

The mean ages at diagnosis of the *CHEK2* variant carriers in patients with HPC were 62.7 years for 1100delC carriers and 64.0 years for I157T carriers. These ages were only marginally different from the mean age of HPC patients with no mutations (65.2 years for both variants; *P*=0.57 for 1100delC and *P*=0.62 for I157T). A similar trend was observed in the cohort of unselected prostate cancer patients (64.6 *vs* 68.7 years; *P*=0.18 for 1100delC and 68.4 *vs* 68.6 years; *P*=0.86 for I157T). The association between the frequency of the two variants and disease phenotype, including tumour WHO-grade, Gleason score, T-, N- and M-stage and PSA value at diagnosis, were also analysed among unselected prostate cancer cases. No significant associations emerged from these analyses (data not shown).

## DISCUSSION

*CHEK2* has been suggested to be a candidate tumour suppressor gene on the basis of the findings that normal function of CHEK2 is involved in DNA-damage respond and some of the mutations identified in Li–Fraumeni families were expected to result in a truncated protein (Bell *et al*, 1999). Subsequently, these findings were supported by the reports concerning identical and additional mutations in patients with Li–Fraumeni syndrome (Lee *et al*, 2001) and breast cancer (Meijers-Heijboer *et al*, 2002; Vahteristo *et al*, 2002). While this manuscript was in preparation, another study was published showing that mutations in *CHEK2* were associated also with prostate cancer risk (Dong *et al*, 2003).

Our results suggest that *CHEK2* 1100delC mutation is associated with positive family history of prostate cancer. The mutation segregated almost completely in all mutation-positive families (Figure 1). In family 351, there were three unaffected men, who carried the variant. Two of them were rather young, about 50 years old (III-2 and III-3), and the third unaffected carrier was 71 years old (II-5). The total PSA values of the unaffected mutation carriers of this family were measured in July 2000. The values were <0.5, 2.2, and 2.4 *μ*g l^−1^ for III-2, III-3 and II-5, respectively. The mean age at diagnosis of prostate cancer in Finland was 71.1 years in 1999 (Finnish Cancer Registry; cancer statistics at http://www.cancerregistry.fi/) and all three affecteds of the family 351 were over 66 years old when diagnosed for prostate cancer, thus the future diagnosis of prostate cancer cannot be ruled out for the healthy carriers of this family. On the other hand, in the four 1100delC-positive families, there were no mutation-negative prostate cancer patients. The association of 1100delC mutation with families that include small number of affected relatives, the most common types of prostate cancer families, implies that the mutation is likely to have a significant contribution to familial prostate cancer at the population level. In addition, I157T seems to be a disease-associated polymorphism at least in the Finnish population. It has a slightly higher frequency among patients with unselected prostate cancer than among control individuals and it is strongly associated with family history of the disease. However, according to the previous reports, the I157T allele does not make a significant contribution to breast cancer susceptibility (Allinen *et al*, 2001; Schutte *et al*, 2003). Therefore, the association with this allele is less conclusive.

Previously, Vahteristo *et al* (2002) reported the strong association of the *CHEK2* 1100delC with breast cancer families that included only two affected patients, suggesting that 1100delC is a low-penetrance genetic alteration. In contrast to our results, Dong *et al* (2003) reported the association of the *CHEK2* mutations (all mutations pooled together) only with sporadic prostate cancer. In addition, they did not observe any association between prostate cancer and I157T variant. The reason why Dong *et al* (2003) did not detect any association with HPC could be due to different sample settings: the families from the USA represent more extreme HPC families than the Finnish families in the present study. In their study two affected members from 149 HPC families with at minimum of three affected men over at least two generations were used. In our recent genome-wide linkage analysis, no positive signals were seen on chromosome 22 (Schleutker *et al*, in press). This is probably due to the selected study material, as only the most extreme families were genotyped, possibly reflecting the same phenomenon as seen in the study of Dong *et al* (2003). Also, the low allele frequencies of *CHEK2* variants (<10%) make this kind of an association almost impossible to detect by linkage analysis.

Dong *et al* (2003) reported a total of 13 different *CHEK2* germline mutations among 400 sporadic prostate cancer patients and 298 individuals with familial prostate cancer. Most of these mutations occurred only once in their study population. The reason why fewer variants were found in our study can possibly be due to the limited sensitivity of the SSCP analysis and the number of screened patients. Most likely, however, the reason is the study population itself. The Finnish population is genetically much more homogeneous than the US population, and therefore it is not surprising that fewer variants were detected.

Taken together, finding of the 1100delC and I157T variants in families with small numbers of affected relatives support the idea that *CHEK2* variants are low-penetrance prostate cancer predisposition alleles that contribute significantly to familial clustering of prostate cancer at the population level, especially in families with small number of affected relatives. However, variants in *CHEK2* gene alone do not explain the familial clustering of prostate cancer in Finland as the majority of families did not have any *CHEK2* alterations. The present results warrant further studies of the role of *CHEK2* variants as a risk factor for prostate cancer in other populations.
